# Characterizations of *bla*_CTX–M–14_ and *bla*_CTX–M–64_ in a clinical isolate of *Escherichia coli* from China

**DOI:** 10.3389/fmicb.2023.1158659

**Published:** 2023-05-18

**Authors:** Mingxing Yang, Dong Liu, Xiaoquan Li, Chuting Xiao, Yingge Mao, Jiaqi He, Jiao Feng, Li Wang

**Affiliations:** ^1^Department of Clinical Laboratory, The First Affiliated Hospital of Henan University, Kaifeng, China; ^2^Institute of Biomedical Sciences, The Key Laboratory of Chemical Biology and Molecular Engineering of Ministry of Education of China, The Key Laboratory of Medical Molecular Cell Biology of Shanxi Province, Shanxi University, Taiyuan, China

**Keywords:** *Escherichia coli*, antimicrobial resistance, ESBLs, CTX-M-14, CTX-M-64

## Abstract

Extended-spectrum beta-lactamase-producing Gram-negative bacteria are common in the community and hospitals. To monitor ESBLs mediated by the CTX-M genotype, we collected clinical ESBL pathogenic strains from a hospital in central China and observed a strain of *Escherichia coli*, namely Ec15103 carrying *bla*_CTX–M–14_, *bla*_CTX–M–64_ and *bla*_TEM–1_, isolated from the blood of a 7-day-old infant in 2015. Strain Ec15103 contains two drug resistance plasmids: pEc15103A, an IncFI-type plasmid that cannot be conjugatively transferred and carries the drug resistance genes *bla*_TEM–1_, *aacC2*, *aadA5*, *sul1*, *mph*(A), *sul2*, *strAB*, and *tetA*(A); and pEc15103B, an IncK2/Z-type plasmid that carries the conjugation transfer gene and *bla*_CTX–M–14_. In addition, *bla*_CTX–M–64_ is located on the chromosome of Ec15103, and it is the first report of pathogen with *bla*_CTX–M–64_ located on its chromosome (the search terms used “blaCTX-M-64” and “chromosome”). *bla*_CTX–M–14_ and *bla*_CTX–M–64_ are carried by IS*Ecp1*-mediated transposon Tn*6503a* and Tn*6502*, respectively. The conjugation transfer ability of pEc15103B was significantly inhibited by zidovudine (AZT) and linoleic acid (LA) and that expression of *bla*_CTX–M–14_, *bla*_CTX–M–64_ and *bla*_TEM–1_ at the mRNA level did not change based on the concentration of cefotaxime or ampicillin. Co-occurrence of *bla*_CTX–M–14_ and *bla*_CTX–M–64_ in a single isolate will enhance the drug resistance of bacteria, and the presence of *bla*_CTX–M–64_ in the chromosome may make the resistance more maintain. This fact will facilitate its dissemination and persistence under different antimicrobial selection pressures. It is essential to prevent these strains from further spreading in a hospital environment.

## Introduction

Refractory infections caused by multidrug-resistant (MDR) bacteria are rapidly increasing worldwide, especially for β-lactamase-producing Gram-negative bacteria (Hussain et al., 2021). These Gram-negative bacteria hydrolyze β-lactams, which renders them resistant to β-lactam antibiotics. According to Ambler’s classification, β-lactamases can be divided into four categories (Ambler et al., 1991): A (serine penicillinases), B (metallo-β-lactamases), C (cephalosporinases), and D (oxacillinases). Extended-spectrum beta-lactamases (ESBLs) are primarily plasmid-mediated, hydrolyzing penicillins, cephalosporins, and monocyclic beta-lactams aztreonam but not cephamycins and carbapenem enzymes. ESBLs can be inhibited by β-lactamase inhibitors, mainly of the class A and D enzymes. Previously, the TEM type and SHV type were the most common genotypes of ESBLs, but since the discovery of the CTX-M β-lactamase, this type has rapidly disseminated worldwide and become the most common ESBL genotype (Bonnet, 2004). It is denoted as CTX-M because it is resistant to cefotaxime but sensitive to ceftazidime (Bauernfeind et al., 1990); it is mainly encoded by plasmids that can be conjugated and transferred. The CTX-M genotype is found in 238 species, and subtypes^1^ are divided into 6 groups according to amino acid heterogeneity: Group 1, Group 2, Group 8, Group 9, Group 25, and KLUC Group (Rossolini et al., 2008).

ESBL-producing Gram-negative bacteria are common in the community and hospitals (Pitout et al., 2005). They cause a relatively high number of clinical infections, which are usually treated with beta-lactamase inhibitors or carbapenems. Therefore, ESBL-producing strains increase the use of carbapenem antibiotics. As this will further accelerate the evolution of bacterial resistance under antibiotic selection pressure (Bevan et al., 2017), monitoring ESBL strains is particularly important.

To monitor ESBLs mediated by the CTX-M genotype, we collected clinical ESBL-producing isolates from a hospital in central China. In this study, *Escherichia coli* Ec15103, which carried both *bla*_CTX–M–14_ and *bla*_CTX–M–64_, was characterized. Moreover, a detailed genetic characterization of pEc15103A and pEc15103B was revealed. *bla*_CTX–M–14_ was located on IncK2/Z-type plasmid pEc15103B, however, *bla*_CTX–M–64_ was harbored in the chromosome.

## Materials and methods

### Bacterial strains

*Escherichia coli* Ec15103 was isolated from the blood of an infected 7-day-old infant at a Chinese teaching hospital in 2015. Bacterial species identification was performed using a Bruker MALDI Biotyper (Bruker Daltonics, Bremen, Germany). *E. coli* Ec600 (*LacZ^–^, Nal*^R^*, Rif*^R^**) was used as the recipient bacterium for plasmid conjugation transfer in this study. Competent *E. coli* EPI300 was used as the recipient bacterium for plasmid chemical transformation. The strains used in this study can be found in Supplementary Table 1.

### ESBL confirmatory test

The ESBL confirmation test used the disc diffusion Antibiotic Sensitivity test (Drieux et al., 2008) and refers to the American Clinical and Laboratory Standards Institute 2020 (CLSI) standard (CLSI, 2020). The zone of inhibition is used to determine the susceptibility of bacteria to an antibiotic. Antimicrobial susceptibility disks (Kangtai Biotechnology, Wenzhou, China) containing ceftazidime (30 μg), cefotaxime (30 μg), ceftazidime/clavulanic acid (30 μg/10 μg), or cefotaxime/clavulanic acid (30 μg/10 μg) were placed on Mueller-Hinton plates (MH plates, Autobio, Zhengzhou, China) coated on the surface with a bacterial suspension (0.5 McFarland turbidity). The size of the inhibition zone was measured after 16–18 h of incubation at 35°C. Measured the inhibition zone around a disk of cephalosporin and around a disk of the same cephalosporin plus clavulanate. Depending on the disk type, a difference of ≥5 mm between the two diameters are considered as indicating ESBL production.

### Plasmid conjugation transfer

Plasmid conjugal transfer experiments were carried out with *E. coli* Ec600 as the recipient and strain Ec15103 as the donor. The recipient and donor bacteria were inoculated in Lysogeny Broth (LB, Solarbio Science & Technology, Beijing, China) and incubated at 37°C and 200 rpm overnight. A total of 50 μL of the overnight cultured bacterial suspension was inoculated in 5 mL of LB broth at 37°C and 200 rpm followed by growth to OD_600_≈1.0, and then 500 μL of donor and recipient bacteria were mixed. The mixture was spotted onto a 1-cm^2^ filter membrane placed on LB nutrient agar (Solarbio Science & Technology, Beijing, China). The plates were incubated at 37°C for 12∼18 h, then the cells were washed from the filter membrane to 1 mL of LB broth. After 1,000-fold dilution, 100 μL was spread on LB nutrient agar medium containing 100 μg/mL ampicillin (Solarbio Science & Technology, Beijing, China) and 750 μg/mL rifampicin (Solarbio Science & Technology, Beijing, China). The colonies after overnight culture at 37°C were transconjugants (Ec15103-Ec600), as verified by PCR.

### Plasmid extraction and transformation

Plasmid DNA of *E. coli* Ec15103 was extracted using a large plasmid extraction kit (Mei5 Biotechnology, Beijing, China), and plasmid transformation was carried out using competent EPI300 cells as the recipient bacteria. First, competent EPI300 cells were taken out from -80°C and quickly placed on ice. After 5 min, the bacteria had thawed, the plasmid DNA of *E. coli* Ec15103 was added, and the mixture was allowed to stand on ice for 30 min. Then, the samples were placed in a water bath at 42°C for 60 s, quickly placed on ice, and allowed to stand for 5 min. Next, 800 μL of LB broth was added to the EP tube and incubated at 37°C for 60 min at 200 rpm, followed by centrifugation at 6,000 rpm for 5 min to collect the bacteria. Approximately 100 μL of the supernatant was retained to resuspend the bacteria and spread the cells on LB medium containing 100 μg/mL ampicillin. The colonies after overnight culture at 37°C were transformants (Ec15103-EPI300-1, Ec15103-EPI300-2), as verified by PCR.

### Detection of resistance genes by PCR and product sequencing

Main resistance genes (*bla*_CTX–M–1G_, *bla*_CTX–M–9G_, and *bla*_TEM–1_) were screened by PCR (for the primers used, see Supplementary Table 2), and the PCR products were sent to Beijing Genomics Institute (BGI, Zhengzhou, China) for sequencing. The sequencing results were compared with the NCBI database to identify the main resistance genes.

### Antimicrobial susceptibility testing

The minimum inhibitory concentrations (MICs) values of 25 antibiotics (see Table 1) in this study were evaluated by the broth microdilution method and determined according to Clinical and Laboratory Standards Institute 2020 guidelines (CLSI, 2020).

**TABLE 1:** Mics of antibiotics and compounds.

Category	Antibiotics	MIC (μ g/mL)/antimicrobial susceptibility					
		Ec15103	Ec600	EPI300	Ec15103 -Ec600	Ec15103- EPI300-1	Ec15103- EPI300-2
β-Lactams	Ampicillin	≥32R	≤8S	≤8S	≥32R	≥32R	≥32R
	Ampicillin/sulbactam	=32/16R	≤2/1S	≤2/1S	=32/16R	=32/16R	=32/16R
	Cefazolin	≥8R	≤2S	≤2S	≥8R	≤2S	≥8R
	Cefuroxime	≥32R	≤8S	≤8S	≥32R	≤8S	≥32R
	Ceftriaxone	≥64R	≤1S	≤1S	≥64R	≤1S	≥64R
	Cefotaxime	≥8R	≤4S	≤4S	≥8R	≤4S	≥8R
	Cefotaxime/clavulanic acid	≤1/4S	≤1/4S	≤1/4S	≤1/4S	≤1/4S	≤1/4S
	Ceftazidime	=16R	≤4S	≤4S	≤4S	≤4S	≤4S
	Ceftazidime/clavulanic acid	≤1/4S	≤1/4S	≤1/4S	≤1/4S	≤1/4S	≤1/4S
	Cefoxitin	≤8S	≤8S	≤8S	≤8S	≤8S	≤8S
	Cefepime	≥32R	≤2S	≤2S	≤2S	≤2S	≤2S
	Piperacillin/tazobactam	≤4/4S	≤4/4S	≤4/4S	≤4/4S	≤4/4S	≤4/4S
	Ticarcillin/clavulanic acid	=64/2I	≤4/2S	≤4/2S	=64/2I	=64/2I	=64/2I
	Cefoperazone/sulbactam	=16/8S	≤2/1S	≤2/1S	=16/8S	≤2/1S	=16/8S
Carbapenems	Meropenem	≤1S	≤1S	≤1S	≤1S	≤1S	≤1S
	Imipenem	≤1S	≤1S	≤1S	≤1S	≤1S	≤1S
Aminoglycosides	Gentamicin	≥16R	≤1S	≤1S	≤1S	≥16R	≥16R
	Amikacin	≤4S	≤4S	≤4S	≤4S	≤4S	≤4S
Fluoroquinolones	Ciprofloxacin	≥4R	≤0.06S	≤0.06S	≤0.06S	≤0.06S	≤0.06S
	Levofloxacin	=4I	≤0.12S	≤0.12S	≤0.12S	≤0.12S	≤0.12S
Others	Polymyxin B	≤2S	≤2S	≤2S	≤2S	≤2S	≤2S
	Co-trimoxazole	≥8/152R	≤0.5/9.5S	=4/76R	≤0.5/9.5S	≥8/152R	≥8/152R
	Nitrofurantoin	≤16S	≤16S	≤16S	≤16S	≤16S	≤16S
	Minocycline	≤4S	≤4S	≤4S	≤4S	≤4S	≤4S
	Chloromycetin	≤8S	≤8S	≤8S	≤8S	≤8S	≤8S

S: susceptible; I: intermediate; R: resistant.

### WGS and analysis

Whole-genome DNA of *E. coli* Ec15103 was extracted with OMEGA D3350 Bacterial DNA Kit (Omega Bio-Tek, Norcross, GA, USA). The *E. coli* strain Ec15103 genome was sequenced using a PacBio Sequel II and DNBSEQ platform at Beijing Genomics Institute (BGI, Shenzhen, China). Four SMRT cells Zero-Mode Waveguide arrays of sequencing were used with the PacBio platform to generate the subread set. PacBio subreads (length < 1 kb) were removed. The program Canu was used for self-correction. Draft genomic unitigs, which are uncontested groups of fragments, were assembled using Canu, a high-quality corrected circular consensus sequence subread set. To improve the accuracy of the genome sequences, GATK^2^ was applied for single-base corrections. Genes were predicted with GeneMarkS™ and RAST and further annotated with BLASTP and BLASTN against the UniProt and NR databases. Annotation of mobile elements was based on the databases ISfinder, INTEGRALL, and Tn Number Registry. Gene organization diagrams were drawn with Inkscape version 0.48.

The genome sequence of strain Ec15103 has been submitted to GenBank: the accession number of the Ec15103 chromosome is CP104274; the accession numbers of pEc15103A and pEc15103B are ON324203 and ON324204, respectively.

### Inhibition of plasmid conjugation transfer

Since zidovudine (AZT, Solarbio Science & Technology, Beijing, China) and linoleic acid (LA, Solarbio Science & Technology, Beijing, China) have different solubilities, we used two methods for inhibiting plasmid conjugation transfer. *E. coli* Ec600 was used as the recipient, and strain Ec15103 was used as the donor. (I) (AZT): The recipient and donor bacteria were inoculated in LB broth at 37°C and 200 rpm overnight. A total of 5 μL of the overnight bacteria was inoculated in 5 mL of LB broth and incubated at 37°C and 200 rpm to OD_600_≈1.0, and then 500 μL of donor and recipient bacteria were mixed. A total of 10 μL of the mixture was spotted on nitrocellulose membrane (4 cm^2^ size, 0.45 μm pore size) placed on LB nutrient agar medium, and the different groups of LB nutrient agar contained different concentrations of AZT. The plates were incubated at 37°C for 12∼18 h, then the cells were washed from the filter membrane to 1 mL of LB broth. After 1,000-fold dilution, 100 μL was spread onto LB nutrient agar medium containing 100 μg/mL ampicillin and 750 μg/mL rifampicin. The colony after 24–28 h of culture at 37°C was considered the transconjugant (Ec15103-Ec600). (II) (LA): Recipient and donor bacteria were inoculated in LB broth and incubated at 37°C and 200 rpm overnight. A total of 5 μL of the overnight bacterial culture was inoculated in 5 mL of LB broth and incubated at 37°C and 200 rpm to OD_600_≈1.0, and then 500 μL of donor and recipient bacteria were mixed. A 10 μL aliquot was added to 5 mL LB broth and cultured overnight at 37°C and 200 rpm. The different groups of LB broth contained different concentrations of LA, which was dissolved in 30% DMSO (Solarbio Science & Technology, Beijing, China). A total of 100 μl of the bacteria were cultured overnight and diluted 100-fold, and taken 100 μL to spread onto LB nutrient agar medium containing 100 μg/mL ampicillin and 750 μg/mL rifampicin. The colony after 24–28 h of culture at 37°C was considered the transconjugant (Ec15103-Ec600). All plasmid conjugation transfer inhibition assays were completed using a minimum of three independent experiments, with three biological replicates per experiment.

### RNA extraction and mRNA expression assays (quantitative real-time PCR)

RNA was extracted from 2 mL of logarithmic phase culture grown in LB broth (OD_600_≈1.0) using a kit (Mei5 Biotechnology, Beijing, China). After reverse transcription real-time quantitative PCR with the SYBR Green method (Pfaffl, 2001), a reaction system with a total volume of 20 μL was established. *E. coli* 16S rRNA was used as a reference gene to observe relative transcription levels of the target genes *bla*_CTX–M–64_, *bla*_CTX–M–14_, and *bla*_TEM–1_ in strains Ec15103, Ec15103-Ec600, and Ec15103-EPI300-1 at different concentrations of cefotaxime (CTX, Solarbio Science & Technology, Beijing, China) or ampicillin (AMP, for the primers used, see Supplementary Table 2). No antibiotics was used as the control group. The 2^–ΔΔ*Ct*^ method, corrected for different primer efficiencies and reference genes, was employed. All mRNA expression assays were completed with a minimum of three independent experiments, with at least three biological replicates per experiment.

### Statistical analyses

Statistical analysis was performed using GraphPad Prism 8 and SPSS software. All data are presented as the mean standard deviation.

## Results

### Characterization of *E. coli* Ec15103

**FIGURE 1:**
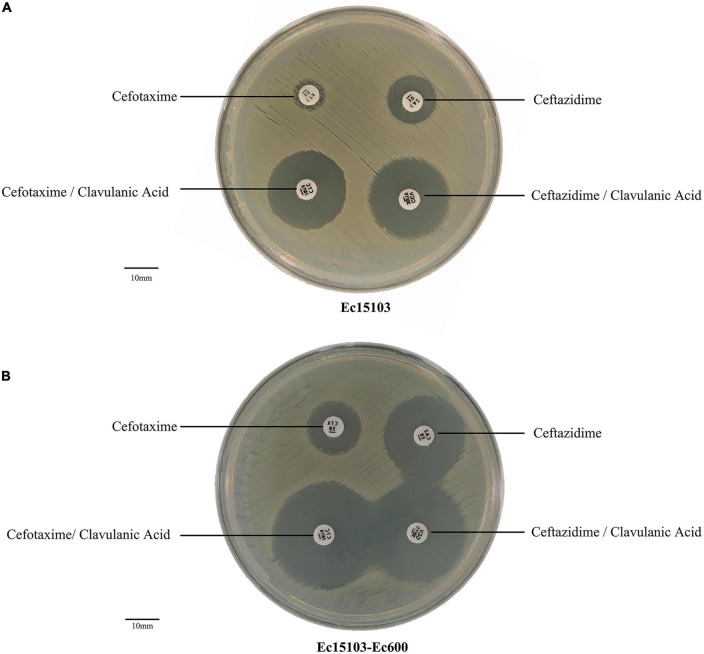
ESBL confirmatory test. **(A)** The ESBL confirmation test of Ec15103. **(B)** The ESBL confirmation test of Ec15103-Ec600. Each experiment was conducted with three parallel replicates, and only one representative figure is shown.

For strain Ec15103, the inhibition zone for cefotaxime was almost 6 mm, and that for cefotaxime/clavulanic acid was 26 mm. The inhibition zone for ceftazidime was 15 mm; that for ceftazidime/clavulanic acid was 23 mm (Figure 1A). The inhibition zone diameter was 5 mm larger than without clavulanic acid, indicating that strain Ec15103 produces ESBL.

**FIGURE 2:**
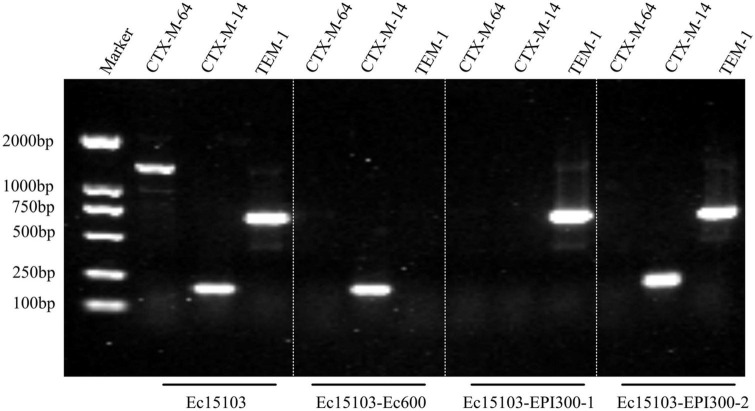
Detection of drug resistance genes by PCR.

As determined by PCR screening and sequencing of the major plasmid-borne ESBL and carbapenem resistance genes, strain Ec15103 harbored *bla*_CTX–*M*–14_, *bla*_CTX–*M*–64_, and *bla*_TEM–1_ but not any of the other genes tested (Figure 2). Plasmid conjugation transfer was carried out using Ec15103 as the donor strain and Ec600 (*LacZ^–^, Nal*^R^*, Rif*^R^**) as the recipient strain. However, only one resistance marker, *bla*_CTX–M–14,_ was transferred from strain Ec15103 to Ec600 through conjugation, generating the transconjugant Ec15103-Ec600 (Figure 2). Additionally, we performed an ESBL confirmation test for Ec15103-Ec600, obtaining an inhibition zone for cefotaxime of 13 mm, for cefotaxime/clavulanic acid of 28 mm, for ceftazidime of 23 mm, and for ceftazidime/clavulanic acid of 30 mm (Figure 1B). As the inhibition zone diameter was 5 mm larger with clavulanic acid than without it, strain Ec15103-Ec600 also shows ESBL enzymatic activity. Both Ec15103 and Ec15103-Ec600 were found to be resistant to ampicillin, ampicillin/sulbactam, cefazolin, cefuroxime, ceftriaxone, and cefotaxime but susceptible to cefoxitin, piperacillin/tazobactam, cefoperazone/sulbactam, meropenem, imipenem, amikacin, polymyxin B, nitrofurantoin, minocycline, and chloramphenicol. Ec15103 was resistant to ceftazidime, cefepime, gentamicin, ciprofloxacin, and cotrimoxazole, whereas Ec15103-Ec600 was not (Table 1).

Furthermore, we extracted plasmid from strain Ec15103 and transformed it into a recipient strain, competent *E. coli* EPI300 (*λ-rpsL (Str*^R^*), trfA*), via chemical transformation. Based on PCR screening, two different transformed strains were detected: Ec15103-EPI300-1 with only one resistance marker, *bla*_*TEM–1*,_ and Ec15103-EPI300-2 with *bla*_TEM–1_ and *bla*_CTX–M–14_ (Figure 2). Their MICs differed from those mentioned above (Table 1). Strain Ec15103-EPI300-1 was found to be resistant to ampicillin, ampicillin/sulbactam, gentamicin and cotrimoxazole, and Ec15103-EPI300-2 to ampicillin, ampicillin/sulbactam, cefazolin, cefuroxime, ceftriaxone, cefotaxime, gentamicin and cotrimoxazole. Both strains were sensitive to ceftazidime, cefepime, and ciprofloxacin.

### Whole-genome sequencing and bioinformatics analysis of strain Ec15103

**FIGURE 3:**
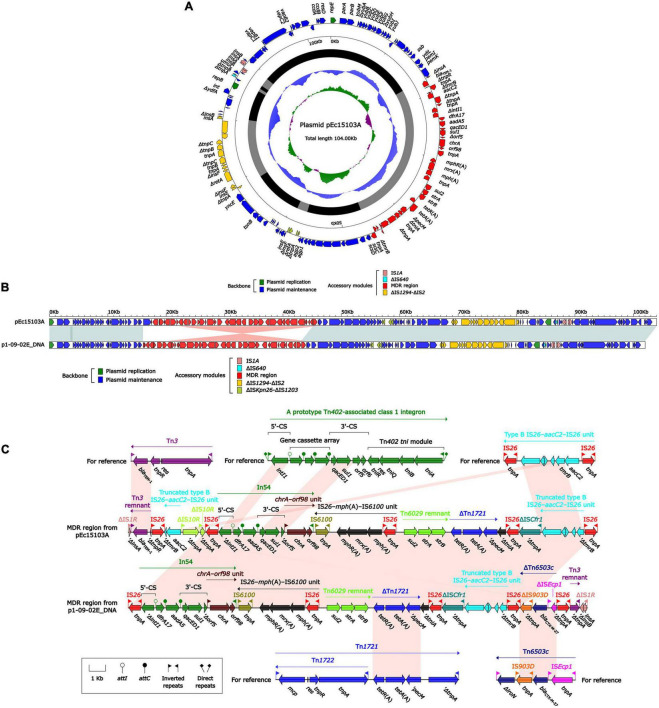
General Features of pEc15103A. **(A)** Schematic map of the sequence of plasmid pEc15103A. Genes are denoted by arrows and colored according to gene function classification. The innermost circle presents the GC skew [(G-C)/(G+C)] with a window size of 500 bp and a step size of 20 bp. The next-to-innermost circle presents the GC content. The backbone and accessory module regions are also shown. **(B)** Linear comparison of the sequenced plasmid pEc15103A. Genes are denoted by arrows colored according to gene function classification. Shaded regions denote regions of homology (>95% nucleotide identity). **(C)** Linear comparison of bla_TEM–1_ genetic surroundings. Genes are denoted by arrows and colored based on gene function classification.

**FIGURE 4:**
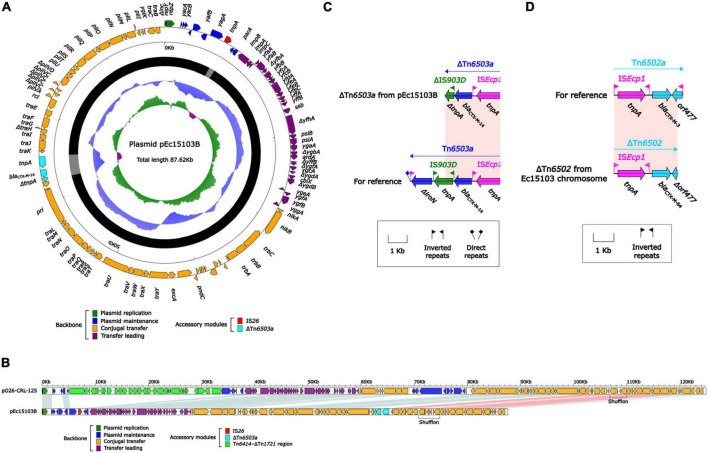
General features of pEc15103B and bla_CTX–M–64_. **(A)** Schematic map of the sequence of plasmid pEc15103B. Genes are denoted by arrows and colored according to gene function classification. The innermost circle presents the GC skew [(G-C)/(G+C)] with a window size of 500 bp and a step size of 20 bp. The next-to-innermost circle presents the GC content. The backbone and accessory module regions are also shown. **(B)** Linear comparison of the sequenced plasmid pEc15103B. Genes are denoted by arrows colored according to gene function classification. Shaded regions denote regions of homology (>95% nucleotide identity). **(C)** Linear comparison of bla_CTX–M–14_ genetic surroundings. Genes are denoted by arrows and colored based on gene function classification. **(D)** Linear comparison of bla_CTX–M–64_ genetic surroundings. Genes are denoted by arrows and colored based on gene function classification.

Whole-genome sequencing (WGS) of strain Ec15103 produced a 4991.97-kb circular chromosome and two plasmids: pEc15103A and pEc15103B. The chromosome has a mean G+C content of 50.6% and consists of 5,121 open reading frames (ORFs). Plasmid pEc15103A is 104.00 kb in length, with an average G+C content of 52.1%, and contains 119 ORFs (Figure 3A). The pEc15103B genome consists of an 87.62-kb circular DNA molecule with an average G+C content of 52.6% and a total of 96 ORFs (Figure 4A). Each plasmid can be divided into backbone and accessory modules. The pEc15103A backbone (Figure 3A) is composed of DNA regions for plasmid replication (*repB* and *repE*) and plasmid maintenance (*parA*, *parB*, *yubM*, *ssb*, etc.). pEc15103A contains five distinct accessory modules (Figure 3A): the MDR region, ΔIS*1294*-ΔIS*2* region, ΔIS*Kpn26*-ΔIS*1203* region, IS*1A*, and ΔIS*640*. The drug resistance genes are all located in the MDR region, including *bla*_TEM–1_, *aacC2*, *aadA5*, *sul1*, *mph*(A), *sul2*, *strAB*, and *tetA*(A). The pEc15103B backbone contains DNA regions for plasmid replication (*repY*, *repZ*), plasmid maintenance (*yacA*, *yacB*, *yagA*, etc.), conjugal transfer (*trs*, *trb*, *pil*, etc.) and transfer (*imp*, *yfa* to *yfh*, *yga* to *ygg*, etc.). pEc15103B harbors only two accessory modules, ΔTn*6503a* and IS*26*, and the drug resistance gene *bla*_CTX–M–14_ is located in the former (Figure 4A).

The overall structure of pEc15103A is most similar to that of p1-09-02E DNA (Mohsin et al., 2020) (GenBank accession number AP022651, 95% query coverage, and 99.99% maximum nucleotide identity). Therefore, p1-09-02E DNA was selected for comparative genomics analysis (Figure 3B). Both pEc15103A and p1-09-02E DNA are IncFI-type plasmids containing two replicons, *RepFIA* and *RepFIB*. Comparative genomic analysis showed that the backbone region of these two plasmids were essentially identical.

The MDR region of pEc15103A contains the following resistance gene modules: a Tn*3* remnant, a truncated type B IS*26*–*aacC2*–IS*26* unit, *In54*, IS*26*–*mph*(A)–IS*6100* unit, a Tn*6029* remnant, and ΔTn*1721* (Figure 3C). Tn*3* is a unit transposon; for pEc15103A, Tn3 is truncated by IS26 to form the Tn3 remnant. The Tn*3* remnant includes a 38-bp IRR, the *bla*_TEM–1_ gene encoding β-lactam resistance, and a truncated *tnpR* (Partridge and Hall, 2005). The IS*26*–*aacC2*–IS*26* unit, firstly observed in plasmid pSTMDT12_L DNA (GenBank accession number AP011958), is a structure containing the aminoglycoside resistance gene *aacC2*, and the core structure of the resistance unit is retained in pEc15103A. In54 is a class 1 integron containing a 5′-conserved segment (5′-CS), variable region, and 3′-conserved segment (3′-CS). The variable region of In*54* contains two gene cassettes carrying *dfrA17*, which encodes quaternary ammonium resistance, and *aadA5*, which encodes aminoglycoside resistance (Changkaew et al., 2014). IS*26*–*mph*(A)–IS*6100* unit is a macrolide resistance vector formed by two insertion sequences, IS*26* and IS*6100*, that combined the macrolide resistance genes *mph*(A), *mrx*, and *mphR*(A) (Partridge, 2011). In pEc15103A, IS*26*–*mph*(A)–*mrx*–*mphR*(A)–IS*6100* is located downstream of In*54*, and this maybe occurred through IS*6100*-mediated recombination (Feng et al., 2017). Tn*6029* is a composite transposon carrying the β-lactamase gene *bla*_TEM–1_, the sulfonamide resistance gene *sul2*, and the streptomycin resistance gene *strAB*; the structure is IS*26*–ΔTn*2*–IS*26*–*repA*–*repC*–*sul2*–*strAB*–IS*26*, where ΔTn*2* is the residue at the 3’ end of Tn*2*, and the structure is *tnpR*–*bla*_TEM–1_–*IRR* (Reid et al., 2015). The structure of the Tn*6029* remnant in pEc15103A is *sul2*–*strAB*. Tn*1721* is a Tn*3* family transposon containing the class A tetracycline resistance module *tetA*(A)–*tetR*(A) (Schmitt et al., 1979), and its linear structure is *IRL*–*mcp*–*res*–*tnpR*–*tnpA*–*IRR-1*–*tetR*(A)–*tetA*(A)–*pecM*–*ΔtnpA*–*IRR-2*. In pEc15103A, the genes associated with Tn1721 transposition were missing, and the remaining structure of Tn*1721* is *tetR*(A)–*tetA*(A)–Δ*pecM*.

The MDR region of p1-09-02E DNA and pEc15103A are similar, and there is only one modular difference: the 4.7-kb 5′-terminal structure of the MDR region of pEc15103A is lost in p1-09-02E DNA, meanwhile, a unique structure (ΔTn*6503c*–IS*26*–Tn*3* remnant–ΔIS*1R*) is added in the 3′-end of MDR region in p1-09-02E DNA. Tn*6503c* is an IS*Ecp1*-mediated transposon and displays an IS*Ecp1*-*bla*_CTX–M–27_-IS*903D*-Δ*iroN* structure (Cheng et al., 2019). For p1-09-02E DNA, Tn*6503c* is truncated by two copies of IS26, and the line structure of ΔTn*6503c* is ΔIS*Ecp1*- *bla*_CTX–M–27_-ΔIS*903D*.

pEc15103B is an IncK2/Z plasmid. In this study, the IncK2/Z reference plasmid pO26-CRL-125 (Venturini et al., 2013) (GenBank accession number KC340960) was included in the comparative genomic analysis (Figure 4B). The results showed that pEc15103B contains the conserved IncK2/Z-type plasmid backbone region. There were two accessory regions in pEc15103B, that was IS26 and ΔTn*6503a*. The *bla*_CTX–M–14_ is located in ΔTn*6503a* (Figure 4C). Tn*6503a* is almost identical to Tn*6503c* except that *bla*_CTX–M–27_ mutates into *bla*_CTX–M–14_. The 3′ end of Tn*6503a* is truncated in pEc15103B, mainly manifesting as deletion of Δ*iroN* and truncation of IS*903D*.

In addition, the chromosome of strain Ec15103 contains the drug resistance gene *bla*_CTX–M–64_, which is located in ΔTn*6502* (Figure 4D). The structure of ΔTn*6502* is IS*Ecp1*-*bla*_CTX–M–3_-*orf477*; in the Ec15103 chromosome, *orf477* in this transposon is truncated, and *bla*_CTX–M–3_ has undergone mutation of individual bases, forming the structure IS*Ecp1*-*bla*_CTX–M–64_-Δ*orf477*.

### Inhibition of plasmid conjugation transfer

**FIGURE 5:**
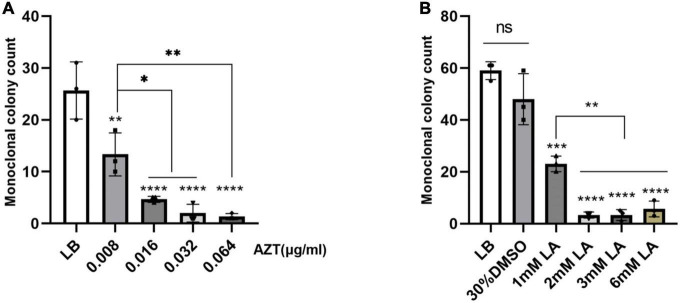
Inhibition of plasmid conjugation transfer experiments. The effect of drugs on plasmid conjugation transfer was evaluated by comparing the monoclonal colony count of conjugants under different drug concentrations. The LB group was the blank control group, and LA was dissolved in 30% DMSO. **(A)** The effect of zidovudine (AZT) on plasmid conjugation transfer; AZT was dissolved in water. **(B)** Effects of linoleic acid (LA) on the conjugative transfer of plasmids; LA was dissolved in 30% DMSO. Data show the mean standard deviation from a minimum of three independent experiments, each with a minimum of three biological replicates. *P < 0.05; **P < 0.01; ***P < 0.001; ****P < 0.0001, statistical method using one-way ANOVA.

Currently, plasmid-mediated intraspecific and interspecific horizontal gene transfer accounts for the majority of the prevalence and spread of antibiotic resistance genes (Alekshun and Levy, 2007; Carattoli, 2013). However, it has been found that some drugs can inhibit plasmid conjugation transfer (Liu et al., 2020). Using strain Ec15103 as the donor strain and Ec600 as the recipient strain, we performed inhibition of plasmid conjugation transfer experiments as described by Buckner et al. (2020). Compared with the LB broth control group, the number of monoclonal clones of the conjugative transfer strain in the AZT drug group decreased in a concentration-dependent manner, even when the concentration was as low as 0.008 μg/mL, the number of monoclonal clones of the conjugative transfer strain was significantly reduced (*P* < 0.01; Figure 5A). When the concentration was 0.064 μg/mL, the number of monoclonal clones of the conjugative transfer strain was close to 0, which was very significant (*P* < 0.0001; Figure 5A). Because LA is insoluble in water, we used 30% DMSO as the solvent, and the results between the 30% DMSO group and the LB broth group were not significantly different (*P* > 0.05; Figure 5B). When the concentration of LA was 1 mM, the number of monoclonal clones of the conjugative transfer strain was significantly reduced (*P* < 0.001; Figure 5B), and the number of monoclonal clones of the conjugative transfer strain was further reduced when the concentration of LA was 2 mM, 3 mM, and 6 mM (*P* < 0.0001; Figure 5B), with a significant difference compared with the 1 mM group (*P* < 0.01; Figure 5B).

### Effect of antibiotics on drug resistance gene expression at the mRNA level

**FIGURE 6:**
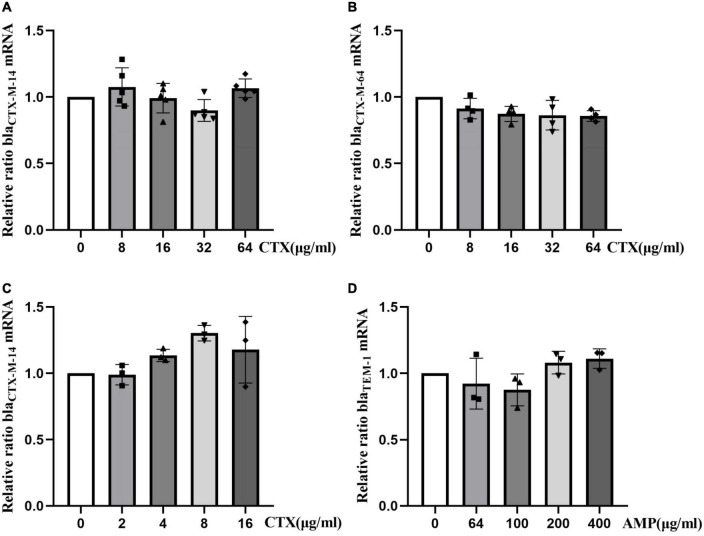
Relative changes in resistance gene mRNA levels in strains Ec15103, Ec15103-Ec600, and Ec15103-EPI300-1 under the influence of antibiotics. **(A,B)** Relative changes in bla_CTX–M–14_ and bla_CTX–M–64_ mRNA levels in strain Ec15103 under different concentrations of cefotaxime (CTX); bla_CTX–M–14_ is present on the Ec15103 plasmid pEc15103B and bla_CTX–M–64_ on the Ec15103 chromosome. **(C)** Relative changes in bla_CTX–M–14_ mRNA levels in strain Ec15103-Ec600 at different concentrations of CTX. bla_CTX–M–14_ is located on the Ec15103-Ec600 conjugate plasmid pEc15103B. **(D)** Relative changes in bla_TEM–1_ mRNA levels in strain Ec15103-EPI300-1 at different concentrations of ampicillin (AMP). bla_TEM–1_ is located on the Ec15103-EPI300-1 transconjugant plasmid pEc15103A. For at least two independent experiments, each with at least three biological replicates, the data shown represent the mean standard deviation using one-way ANOVA.

To investigate the effect of antibiotics on expression of resistance genes at the mRNA level, different concentrations of antibiotics were used, and expression of resistance genes was determined by RT-qPCR. Analysis of *bla*_CTX–M–14_ and *bla*_CTX–M–64_ mRNA levels of strain Ec15103 at different concentrations of CTX showed that expression of *bla*_CTX–M–14_ and *bla*_CTX–M–64_ did not differ significantly in the presence of CTX (Figures 6A, B). Similarly, expression of *bla*_CTX–M–14_ did not change substantially in strain Ec15103-Ec600 in the presence of CTX (Figure 6C), nor did *bla*_TEM–1_ mRNA levels in strain Ec15103-EPI300-1 at different concentrations of AMP (Figure 6D).

## Discussion

In this study, *E. coli* Ec15103 was isolated from the blood of a 7-day-old infant in 2015, and Ec15103 harbored two CTX-M genes: *bla*_CTX–M–14_ and *bla*_CTX–M–64_. Genome sequencing denoted two drug resistance plasmids: pEc15103A is an IncFI-type plasmid that cannot be conjugatively transferred and contains the drug resistance genes *bla*_TEM–1_, *aacC2*, *aadA5*, *sul1*, *mph*(A), *sul2*, *strAB*, and *tetA*(A); pEc15103B contains the conjugation transfer gene, is an IncK2/Z-type plasmid and contains *bla*_CTX–M–14_. In pEc15103B, *bla*_CTX–M–14_ is located in ΔTn*6503a* with a structure of IS*Ecp1*-*bla*_CTX–M–14_-ΔIS*903D*. *bla*_CTX–M–64_ was located on the chromosome of strain Ec15103, and it is the first report of pathogen with *bla*_CTX–M–64_ located on its chromosome (the search terms used “blaCTX-M-64” and “chromosome”).

In plasmid conjugation transfer inhibition experiments, the conjugation transfer ability of plasmid pEc15103B was significantly inhibited by AZT and LA, which is consistent with the results of Buckner et al. (2020). In general, conjugative transfer of plasmids contributes significantly to the spread of drug resistance genes (Grohmann et al., 2003; Harrison and Brockhurst, 2012). In the post-antibiotic era, it seems reasonable to curb the spread of drug resistance genes and slow the pace of superbug production by inhibiting plasmid conjugation transfer, as the human intestine is a natural library for drug resistance gene spread (Huddleston, 2014). AZT was the first antiretroviral agent to be licensed for treating human immunodeficiency virus (HIV) infection (Rachlis, 1990), and 0.008 μg/mL is much lower than the dose of the drug in AIDS treatment. However, it can significantly inhibit the conjugative transfer of the plasmid. LA, which is an essential nutrient (Whelan and Fritsche, 2013), is very effective in inhibiting the conjugation and transfer of plasmids, and is an excellent choice. Nevertheless, these drugs may not be effective for the treatment of infection and will increase the patient’s treatment burden, and there is no animal study on their inhibitory effect.

In addition, we sought to determine whether the expression of these resistance genes increases with an increase in antibiotic concentration. Therefore, we conducted qPCR experiments to verify the relationship between expression of drug-resistance genes and the concentration of antibiotics at the mRNA level. Under the conditions of different concentrations of cefotaxime, mRNA expression levels of the genes *bla*_CTX–M–14_ and *bla*_CTX–M–64_ in strains Ec15103 and Ec15103-Ec600 did not change significantly. Similarly, under the effect of ampicillin, expression at the mRNA level of the *bla*_TEM–1_ gene of strain Ec15103-EPI300-1 was also unaffected. This is different from the description by Kjeldsen et al. (2015), probably because of differences between strains, and some studies had noted that relative expression at the mRNA level and the protein level of the *bla*_CTX–M_ gene differ (Geyer et al., 2016).

Due to the presence of *bla*_CTX–M–64_ on the chromosome, strain Ec15103 exhibited resistant to ceftazidime; conversely, other conjugated and transformed strains were sensitive. *bla*_CTX–M–64_ was first reported in 2009 and found on a Shigella drug resistance plasmid in Japanese patients with diarrhea after travel to China (Nagano et al., 2009). It is considered to be a hybrid chimera of *bla*_CTX–M–15_ (CTX-M-1G) and *bla*_CTX–M–14_ (CTX-M-9G) and shows strong ceftazidime hydrolysis activity. There were relatively few subsequent reports of pathogenic bacteria carrying the *bla*_CTX–M–64_ resistance gene, and in most cases, it was found on plasmids. At present, it is generally believed that the CTX-M gene derive from the chromosome of *Kluyvera* spp (Poirel et al., 2002) and that it became incorporated on a plasmid through the action of mobile elements such as transposons and integrons. Therefore, it is generally believed that the CTX-M resistance genes in other bacteria are mainly located on plasmids (D’Andrea et al., 2013). However, there are an increasing number of reports of chromosomally located CTX-M genes (most of which are *bla*_CTX–M–15_ and *bla*_CTX–M–14_) recently (Irrgang et al., 2017; Zheng et al., 2021). Therefore, as the CTX-M drug resistance gene derives from the chromosome, it may again be inserted into the bacterial chromosome. In addition to *E. coli*, *Klebsiella pneumoniae* (Coelho et al., 2010), *Acinetobacter baumannii* (Potron et al., 2011), *Proteus mirabilis* (Song et al., 2011), and *Salmonella* (Zhang et al., 2019) all harbor CTX-M gene on the chromosome. This also demonstrates that insertion of *bla*_CTX–M_ into the chromosome by a plasmid under the action of a transposon is not a random event. Indeed, bacterial resistance may be evolving in this direction. Because the presence of plasmids is not without cost to bacteria, those containing plasmids often have a relatively long replication cycle (Harrison and Brockhurst, 2012), and the plasmids may be lost during passage and reproduction. Once the resistance gene is inserted into the chromosome, there is no longer a cost, and the resistance gene will continue to replicate with each division cycle, presenting another challenge for antibiotic treatment.

## Data availability statement

The datasets presented in this study can be found in online repositories. The names of the repository/repositories and accession number(s) can be found below: https://www.ncbi.nlm.nih.gov/genbank/, CP104274; https://www.ncbi.nlm.nih.gov/genbank/, ON324203; https://www.ncbi.nlm.nih.gov/genbank/, ON324204.

## Ethics statement

Written informed consent was obtained from the individual(s) for the publication of any potentially identifiable images or data included in this article.

## Author contributions

LW and JF conceived the study. MY and JH performed the experiments. LW and MY analyzed the data. XL, CX, DL, and YM contributed reagents, materials, and analysis tools. LW, JF, and MY wrote the manuscript. All authors listed have made a substantial, direct, and intellectual contribution to the work, and approved it for publication.

## Footnotes

1
https://card.mcmaster.ca/ontology/36025

2
https://www.broadinstitute.org/gatk/

## References

[B1] Alekshun M. N. Levy S. B. (2007). Molecular mechanisms of antibacterial multidrug resistance. *Cell* 128 1037–1050. 10.1016/j.cell.2007.03.004 17382878

[B2] Ambler R. P. Coulson A. F. Frère J. M. Ghuysen J. M. Joris B. Forsman M. (1991). A standard numbering scheme for the class a beta-lactamases. *Biochem. J.* 276(Pt 1) 269–270. 2039479 10.1042/bj2760269 PMC1151176

[B3] Bauernfeind A. Grimm H. Schweighart S. (1990). A new plasmidic cefotaximase in a clinical isolate of *Escherichia coli*. *Infection* 18 294–298. 10.1007/BF01647010 2276823

[B4] Bevan E. R. Jones A. M. Hawkey P. M. (2017). Global epidemiology of CTX-M β-lactamases: Temporal and geographical shifts in genotype. *J. Antimicrob. Chemother.* 72 2145–2155. 10.1093/jac/dkx146 28541467

[B5] Bonnet R. (2004). Growing group of extended-spectrum beta-lactamases: The CTX-M enzymes. *Antimicrob. Agents Chemother.* 48 1–14. 10.1128/AAC.48.1.1-14.2004 14693512 PMC310187

[B6] Buckner M. M. C. Ciusa M. L. Meek R. W. Moorey A. R. Mccallum G. E. Prentice E. L. (2020). HIV drugs inhibit transfer of plasmids carrying extended-spectrum β-lactamase and carbapenemase genes. *mBio* 11:e03355-19. 10.1128/mBio.03355-19 PMC7042701 32098822

[B7] Carattoli A. (2013). Plasmids and the spread of resistance. *Int. J. Med. Microbiol.* 303 298–304. 23499304 10.1016/j.ijmm.2013.02.001

[B8] Changkaew K. Utrarachkij F. Siripanichgon K. Nakajima C. Suthienkul O. Suzuki Y. (2014). Characterization of antibiotic resistance in *Escherichia coli* isolated from shrimps and their environment. *J. Food Protect.* 77 1394–1401. 10.4315/0362-028X.JFP-13-510 25198603

[B9] Cheng Q. Jiang X. Xu Y. Hu L. Luo W. Yin Z. (2019). Type 1, 2, and 1/2-hybrid IncC plasmids from China. *Front. Microbiol.* 10:2508. 10.3389/fmicb.2019.02508 31803147 PMC6872532

[B10] CLSI (2020). *Performance Standards for Antimicrobial Susceptibility Testing: CLSI Supplement M100*, 30th Edn. Wayne, PA: Clinical and Laboratory Standards Institute.

[B11] Coelho A. González-López J. J. MIRó E. Alonso-TARRéS C. Mirelis B. Larrosa M. N. (2010). Characterisation of the CTX-M-15-encoding gene in *Klebsiella pneumoniae* strains from the Barcelona metropolitan area: Plasmid diversity and chromosomal integration. *Int. J. Antimicrob. Agents* 36 73–78. 10.1016/j.ijantimicag.2010.03.005 20392607

[B12] D’Andrea M. M. Arena F. Pallecchi L. Rossolini G. M. (2013). CTX-M-type β-lactamases: A successful story of antibiotic resistance. *Int. J. Med. Microbiol.* 303 305–317. 23490927 10.1016/j.ijmm.2013.02.008

[B13] Drieux L. Brossier F. Sougakoff W. Jarlier V. (2008). Phenotypic detection of extended-spectrum beta-lactamase production in *Enterobacteriaceae*: Review and bench guide. *Clin. Microbiol. Infect.* 14(Suppl. 1) 90–103. 10.1111/j.1469-0691.2007.01846.x 18154532

[B14] Feng J. Yin Z. Zhao Q. Zhao Y. Zhang D. Jiang X. (2017). Genomic characterization of novel IncFII-type multidrug resistant plasmids p0716-KPC and p12181-KPC from *Klebsiella pneumoniae*. *Sci. Rep.* 7:5830. 10.1038/s41598-017-06283-z 28725038 PMC5517477

[B15] Geyer C. N. Fowler R. C. Johnson J. R. Johnston B. Weissman S. J. Hawkey P. (2016). Evaluation of CTX-M steady-state mRNA, mRNA half-life and protein production in various STs of *Escherichia coli*. *J. Antimicrob. Chemother.* 71 607–616. 10.1093/jac/dkv388 26612874 PMC4743699

[B16] Grohmann E. Muth G. Espinosa M. (2003). Conjugative plasmid transfer in gram-positive bacteria. *Microbiol. Mol. Biol. Rev.* 67 277–301. 12794193 10.1128/MMBR.67.2.277-301.2003 PMC156469

[B17] Harrison E. Brockhurst M. A. (2012). Plasmid-mediated horizontal gene transfer is a coevolutionary process. *Trends Microbiol.* 20 262–267. 10.1016/j.tim.2012.04.003 22564249

[B18] Huddleston J. R. (2014). Horizontal gene transfer in the human gastrointestinal tract: Potential spread of antibiotic resistance genes. *Infect. Drug Resist.* 7 167–176. 25018641 10.2147/IDR.S48820 PMC4073975

[B19] Hussain H. I. Aqib A. I. Seleem M. N. Shabbir M. A. Hao H. Iqbal Z. (2021). Genetic basis of molecular mechanisms in β-lactam resistant gram-negative bacteria. *Microb. Pathog.* 158:105040. 10.1016/j.micpath.2021.105040 PMC8445154 34119627

[B20] Irrgang A. Falgenhauer L. Fischer J. Ghosh H. Guiral E. Guerra B. (2017). CTX-M-15-producing *E. coli* isolates from food products in germany are mainly associated with an IncF-type plasmid and belong to two predominant clonal *E. coli* lineages. *Front. Microbiol.* 8:2318. 10.3389/fmicb.2017.02318 29209306 PMC5702323

[B21] Kjeldsen T. S. Overgaard M. Nielsen S. S. Bortolaia V. Jelsbak L. Sommer M. (2015). CTX-M-1 β-lactamase expression in *Escherichia coli* is dependent on cefotaxime concentration, growth phase and gene location. *J. Antimicrob. Chemother.* 70 62–70. 25182062 10.1093/jac/dku332

[B22] Liu Y. Tong Z. Shi J. Jia Y. Yang K. Wang Z. (2020). Correlation between exogenous compounds and the horizontal transfer of plasmid-borne antibiotic resistance genes. *Microorganisms* 8:1211. 10.3390/microorganisms8081211 32784449 PMC7463591

[B23] Mohsin M. Tanaka K. Kawahara R. Kondo S. Noguchi H. Motooka D. (2020). Whole-genome sequencing and comparative analysis of the genomes of *Bacteroides* thetaiotaomicron and *Escherichia coli* isolated from a healthy resident in Vietnam. *J. Glob. Antimicrob. Resist.* 21 65–67. 10.1016/j.jgar.2020.02.034 32200128

[B24] Nagano Y. Nagano N. Wachino J. Ishikawa K. Arakawa Y. (2009). Novel chimeric beta-lactamase CTX-M-64, a hybrid of CTX-M-15-like and CTX-M-14 beta-lactamases, found in a *Shigella* sonnei strain resistant to various oxyimino-cephalosporins, including ceftazidime. *Antimicrob. Agents Chemother.* 53 69–74. 10.1128/AAC.00227-08 18955524 PMC2612187

[B25] Partridge S. R. (2011). Analysis of antibiotic resistance regions in Gram-negative bacteria. *FEMS Microbiol. Rev.* 35 820–855. 21564142 10.1111/j.1574-6976.2011.00277.x

[B26] Partridge S. R. Hall R. M. (2005). Evolution of transposons containing blaTEM genes. *Antimicrob. Agents Chemother.* 49 1267–1268. 15728947 10.1128/AAC.49.3.1267-1268.2005 PMC549287

[B27] Pfaffl M. W. (2001). A new mathematical model for relative quantification in real-time RT-PCR. *Nucleic Acids Res.* 29:e45. 10.1093/nar/29.9.e45 PMC55695 11328886

[B28] Pitout J. D. Nordmann P. Laupland K. B. Poirel L. (2005). Emergence of *Enterobacteriaceae* producing extended-spectrum beta-lactamases (ESBLs) in the community. *J. Antimicrob. Chemother.* 56 52–59. 10.1093/jac/dki166 15917288

[B29] Poirel L. KäMPFER P. Nordmann P. (2002). Chromosome-encoded Ambler class A beta-lactamase of Kluyvera georgiana, a probable progenitor of a subgroup of CTX-M extended-spectrum beta-lactamases. *Antimicrob. Agents Chemother.* 46 4038–4040. 10.1128/AAC.46.12.4038-4040.2002 12435721 PMC132763

[B30] Potron A. Munoz-Price L. S. Nordmann P. Cleary T. Poirel L. (2011). Genetic features of CTX-M-15-producing *Acinetobacter baumannii* from Haiti. *Antimicrob. Agents Chemother.* 55 5946–5948. 10.1128/AAC.05124-11 21930877 PMC3232807

[B31] Rachlis A. R. (1990). Zidovudine (Retrovir) update. *Cmaj* 143 1177–1185. 2224694 PMC1452857

[B32] Reid C. J. Chowdhury P. R. Djordjevic S. P. (2015). Tn6026 and Tn6029 are found in complex resistance regions mobilised by diverse plasmids and chromosomal islands in multiple antibiotic resistant *Enterobacteriaceae*. *Plasmid* 80 127–137. 10.1016/j.plasmid.2015.04.005 25917547

[B33] Rossolini G. M. D’Andrea M. M. Mugnaioli C. (2008). The spread of CTX-M-type extended-spectrum beta-lactamases. *Clin. Microbiol. Infect.* 14(Suppl. 1) 33–41. 18154526 10.1111/j.1469-0691.2007.01867.x

[B34] Schmitt R. Bernhard E. Mattes R. (1979). Characterisation of Tn1721, a new transposon containing tetracycline resistance genes capable of amplification. *Mol. General Genet.* 172 53–65. 10.1007/BF00276215 377024

[B35] Song W. Kim J. Bae I. K. Jeong S. H. Seo Y. H. Shin J. H. (2011). Chromosome-encoded AmpC and CTX-M extended-spectrum β-lactamases in clinical isolates of *Proteus mirabilis* from Korea. *Antimicrob Agents Chemother.* 55 1414–1419. 21282448 10.1128/AAC.01835-09 PMC3067170

[B36] Venturini C. Hassan K. A. Roy C. Paulsen I. T. Walker M. J. Djordjevic S. P. (2013). Sequences of two related multiple antibiotic resistance virulence plasmids sharing a unique IS26-related molecular signature isolated from different *Escherichia coli* pathotypes from different hosts. *PLoS One* 8:e78862. 10.1371/journal.pone.0078862 24223859 PMC3817090

[B37] Whelan J. Fritsche K. (2013). Linoleic acid. *Adv. Nutr.* 4 311–312. 23674797 10.3945/an.113.003772 PMC3650500

[B38] Zhang C. Z. Ding X. M. Lin X. L. Sun R. Y. Lu Y. W. Cai R. M. (2019). The emergence of chromosomally located bla (CTX-M-55) in *Salmonella* from foodborne animals in China. *Front. Microbiol.* 10:1268. 10.3389/fmicb.2019.01268 31231347 PMC6560199

[B39] Zheng W. Yue M. Zhang J. Ruan Z. (2021). Coexistence of two bla(CTX-M-14) genes in a bla(NDM-5)-carrying multidrug-resistant *Escherichia coli* strain recovered from a bloodstream infection in China. *J. Glob. Antimicrob. Resist.* 26 11–14. 10.1016/j.jgar.2021.05.002 34023530

