# Supplementary material for: Characterizations of blaCTX–M–14 and blaCTX–M–64 in a clinical isolate of Escherichia coli from China
Source: Front Microbiol. 2023 May 18;14:1158659. doi: 10.3389/fmicb.2023.1158659 (PMC10464524; doi:10.3389/fmicb.2023.1158659)
Supplement: Supplementary file 1 [file Table_1.DOCX]

Supplementary Material

**Supplementary Tables**

**Table S1. Strains used in this study.**

| **Strain** | **Genotype** | **References** |
| --- | --- | --- |
| Ec15103 | *bla*CTX-M-64 、*bla*TEM-1 、*bla*CTX-M-14 | This work |
| Ec600 | *LacZ^-^、Nal^R^、Rif^R^* | (Cai et al., 2008) |
| EPI300 | *λ- rpsL (Str^R^) 、trfA* | (Card et al., 2014, Chen et al., 2006) |
| Ec15103-EC600 | *bla*CTX-M-14 | This work |
| Ec15103-EPI300-1 | *bla*TEM-1 | This work |
| Ec15103-EPI300-2 | *bla*TEM-1、*bla*CTX-M-14 | This work |

**Table S2. Primers used in this study.**

| **PRIMER** | **PRIMER SEQUENCE** | **PRODUCT (bp)** | **APPLICATION** |
| --- | --- | --- | --- |
| bla_CTX-M-1G_ | forward：5'-GGAGTGTATGAAAAATGTCTGG-3' | 1376 | sequencing + proof of conjugation |
|  | reverse：5'-CGTGCTGTCGCTGGATACC-3' |  |  |
| bla_CTX-M-9G_ | forward：5'-GGAGTGTATGAAAAATGTCTGG-3' | 1301 | sequencing |
|  | reverse：5'-TGGAGCCACGGTTGATGAG-3' |  |  |
| bla_TEM-1_ | forward：5'-AATGATACCGCGAGACCCAC-3' | 716 | Sequencing + proof of conjugation |
|  | reverse：5'-TTCGTGTCGCCCTTATTCCC-3' |  |  |
| bla_CTX-M-14_ | forward：5'-CAAAGAGAGTGCAACGGATG-3' | 205 | qPCR + proof of conjugation |
|  | reverse：5'-ATTGGAAAGCGTTCATCACC-3' |  |  |
| E.coli-16s | forward：5'-TGCCTGATGGAGGGGGATAA-3' | 166 | qPCR |
|  | reverse：5'-TCAGACCAGCTAGGGATCGT-3' |  |  |
| bla_CTX-M-64_ | forward：5'-AACTTGCCGAATTAGAGCGG-3' | 168 | qPCR |
|  | reverse：5'-GCTTTTGCGTTTCACTCTGC-3' |  |  |
| bla_TEM-1_ | forward：5'-AATAAACCAGCCAGCCGGAA-3' | 200 | qPCR |
|  | reverse：5'-GGGAACCGGAGCTGAATGAA-3' |  |  |

**References:**

CAI, J. C., ZHOU, H. W., ZHANG, R. & CHEN, G.-X. 2008. Emergence of Serratia marcescens, Klebsiella pneumoniae, and Escherichia coli Isolates possessing the plasmid-mediated carbapenem-hydrolyzing beta-lactamase KPC-2 in intensive care units of a Chinese hospital. *Antimicrobial agents and chemotherapy,* 52**,** 2014-8.

CARD, R. M., WARBURTON, P. J., MACLAREN, N., MULLANY, P., ALLAN, E. & ANJUM, M. F. 2014. Application of microarray and functional-based screening methods for the detection of antimicrobial resistance genes in the microbiomes of healthy humans. *PloS one,* 9**,** e86428.

CHEN, Y.-T., SHU, H.-Y., LI, L.-H., LIAO, T.-L., WU, K.-M., SHIAU, Y.-R., YAN, J.-J., SU, I.-J., TSAI, S.-F. & LAUDERDALE, T.-L. 2006. Complete nucleotide sequence of pK245, a 98-kilobase plasmid conferring quinolone resistance and extended-spectrum-beta-lactamase activity in a clinical Klebsiella pneumoniae isolate. *Antimicrobial agents and chemotherapy,* 50**,** 3861-6.
